# Evaluation of an event-based camera for time-resolved imaging of primary atomization in an air-assist atomizer

**DOI:** 10.1007/s00348-025-04009-w

**Published:** 2025-04-03

**Authors:** Kuppuraj Rajamanickam, Yannis Hardalupas

**Affiliations:** Department of Mechanical Engineering, Imperial College London, London, UK

## Abstract

The current work discusses the demonstration of an event-based (EB) camera for time-resolved imaging (10,000 frames/sec) of the primary atomization of a canonical air-assist atomizer. Experiments were performed simultaneously with conventional high-speed and event-based cameras, enabling us to quantitatively assess the performance of event-based cameras in spray imaging (particularly near-field liquid jet breakup) applications. Three atomization breakup regimes are considered: columnar, bag, and multimode. Dynamic mode decomposition (DMD) was implemented to analyze the acquired instantaneous time-dependent images from both cameras and assess their performance in extracting turbulence statistics of the primary atomization. The computed DMD frequency spectrum and spatial modes of liquid breakup characteristics from the images recorded from both cameras are comparable, highlighting the potential of event-based cameras in extracting coherent structures in the primary atomization zone and their spectral contents. However, in some instances, the EB camera underpredicts the DMD modes compared to high-speed cameras, and the reasons for these discrepancies were explained. Finally, the limitations (e.g., event saturation) of event-based cameras in the context of primary atomization imaging were also discussed.

**Supplementary Information:**

The online version contains supplementary material available at 10.1007/s00348-025-04009-w.

## Introduction

Time-resolved imaging becomes crucial in resolving turbulence characteristics during primary atomization, whose time scales are in the order of milliseconds. Such measurements are necessary to understand the spray formation process, where the gas–liquid interface involves complex interactions between the eddies of multiple lengths and time scales (Lasheras and Hopfinger 2000) (Marmottant and Villermaux 2004) (Rajamanickam and Basu 2017). Advancements in digital camera sensor technology enable the imaging of flows at high frame rates (e.g., time-resolved PIV). However, massive data storage requirements and high costs remain a bottleneck, especially for 3D imaging requiring multiple cameras. This motivates the development of alternate time-resolved imaging devices.

Event-based (EB) cameras are emerging as an alternative approach to high-speed cameras in many applications (Rebecq et al. 2019) (Gallego et al. 2020). Carver Mead and Misha Mahowald originally proposed the EB camera in early 1990 at Caltech. It was initially developed by mimicking the eye’s retina, and hence, it is also classically referred to as a *neuromorphic camera, dynamic vision sensor, silicon retina,* etc. Unlike traditional frame-based cameras, event cameras record only changes in light intensity (*I*) in the scene. Each pixel on the event camera detector responds individually to light intensity changes, continuously producing an asynchronous stream of events. Noticeably, the EB camera can detect intensity changes at a high temporal resolution of about 1 microsecond (Lichtsteiner et al. 2008), making it a potential alternative to the traditional high-speed camera. Moreover, the intensity changes are recorded in the form of binary numbers 1 (increasing light intensity) and 0 (decreasing light intensity), significantly reducing the data size compared to conventional high-speed cameras. Over the years, EB cameras have evolved from a pixel resolution of 0.16 MPix (Lichtsteiner et al. 2008) to 1 MPix. Noteworthy is that nowadays many commercial providers (e.g., Prophesee) offer evaluation board-level sensors, allowing the users to customize them. This broadens the application domains of EB cameras, ranging from robotics to fluid dynamics.

The first demonstration of an EB camera in fluid dynamics application was done by Drazen et al. (2011). Their work showcased time-resolved particle tracking velocimetry (PTV) in solid–liquid two-phase flows using an EB camera and continuous wave (CW) laser. Later, Ni et al. (2012) demonstrated the EB camera for real-time particle detection in a microchannel using the Hough circle transform. Borer et al. (2017) employed three EB cameras (Davis 128) to reconstruct the time-resolved three-dimensional velocity field in the relatively large field of view. Their system employed a halogen lamp instead of a high-power laser as an illumination source. Howell et al. (2020) showed the potential of an EB camera (Prophesee-CSD3SHCD) coupled with a microscope for particle detection and subsequent microparticle tracking velocimetry (µ-PTV) in a rectangular microfluidic channel with a cross section of 360 µm × 60 µm. Notably, they have demonstrated the implementation of µ-PTV using an EB camera without a high-power laser. It should be noted that eliminating such high-power laser in a µ-PTV system would significantly reduce the capital cost. Rusch and Rösgen (2023) proposed the novel TracAER system utilized multiple event cameras to obtain a three-dimensional velocity field in relatively large measurement volumes of ~ 3 × 1 × 1 m^3^ with a high temporal resolution of 10,000 frames/s. Like Howell et al. (2020), their system also employed low-cost LEDs as an illumination source instead of a high-power laser. More recently, Willert and Klinner (2022a) and Willert (2023) explored the potential and limitations (e.g., pixel latency) of PIV implementation using EB camera to a greater extent by comparing its results with the traditional PIV. Noteworthy is that Willert (2023) quantitatively assessed and demonstrated the pulsed illumination in velocimetry applications to address some of the shortcomings (e.g., pixel latency in highly dense particles) in modern-day EB cameras.

Thus, overall, the above discussions delineate the potential of EB cameras in time-resolved imaging of fluid flows. However, to the best of the authors’ knowledge, such cameras have not been tested extensively for spray imaging. Henceforth, the present work assesses event-based cameras for high-speed imaging of the primary atomization process in canonical air-assist atomizers.

## Event-based imaging—working principle

**Fig. 1:**
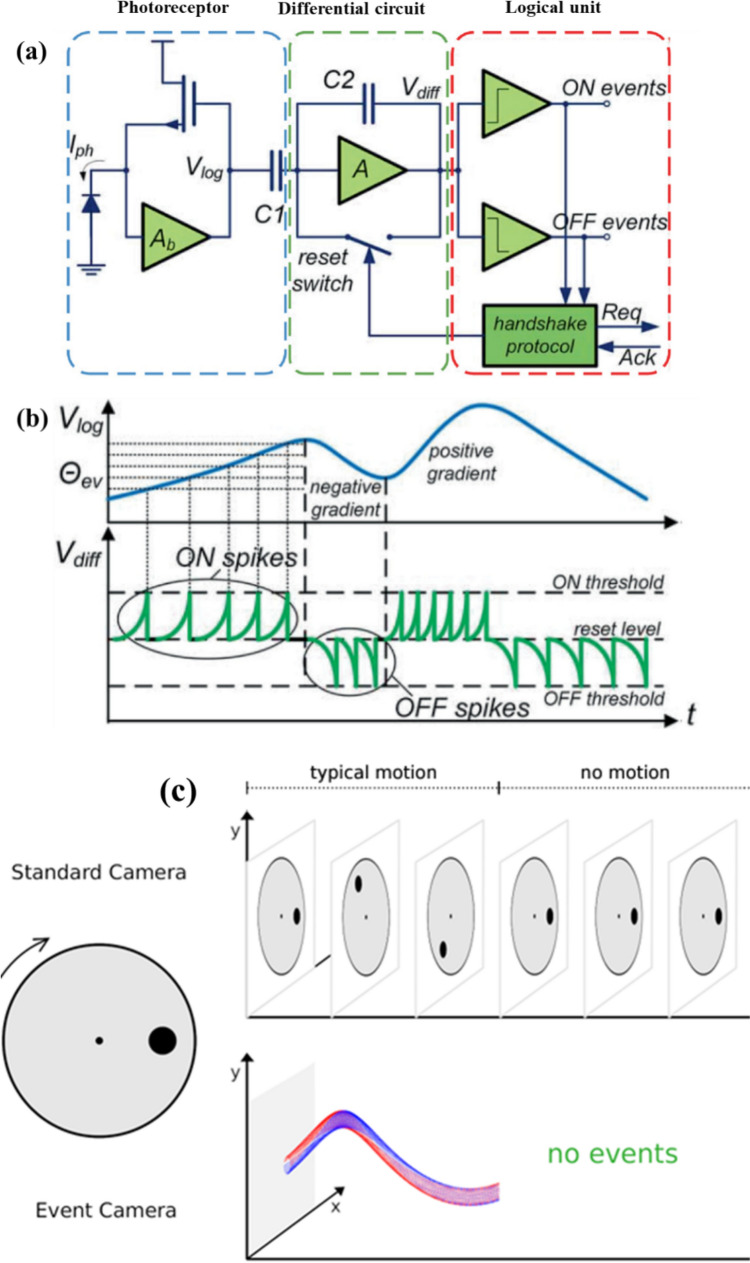
**a** Pixel architecture of event camera; **b** working principle of contrast detector (Shao et al. 2023); **c** comparison between event and frame cameras (Bull and Zhang 2021)

The simplified view of the event camera’s pixel architecture and its operation is illustrated in Fig. 1** a**. Unlike frame-based cameras, each pixel in the event camera responds individually to the local luminescence changes in the scene. Each pixel in the event camera comprises a photoreceptor, differential circuit (contrast detector), and logical unit. First, the photocurrent generated by induced photons at the pixel is converted into the voltage (V). Then, the logarithm of the voltage is fed into the differential circuit to detect the contrast changes. Finally, the logical unit differentiates the detected contrast changes into positive (ON) and negative (OFF) events based on the light intensity. As shown in Fig. 1b, positive and negative events represent the change in light intensity from darker to brighter and brighter to darker, respectively.

Figure 1c compares the output of the spinning disk with the dot captured with conventional frame and event-based cameras. The frame camera captures the entire disk and generates an image based on the sampling rate at discrete time steps. On the other hand, the contrast detector in the event camera continuously scans the temporal variations in the incident light $I(t)$. It generates an event only when the $I(t)$ differs (usually > 10%) from the reference threshold${(I}_{ref})$. It should be noted that ${I}_{ref}$ can be user-defined in most event-based cameras. In the above-shown example, as the disk rotates, the light intensity changes whenever the dot traverses the pixels; hence, the event camera captures only the dot’s motion. Moreover, the output from the event camera is continuous in time. The event camera stops generating events when the disk stops, while the frame camera continues to capture images (see Fig. 1c). In other words, an event camera captures only objects moving in the scene. This is an incredibly beneficial feature in fluid flow imaging, especially near-wall flows, where reflections/glare from the wall significantly affect the SNR. The output data stream of the EB camera is in the format of $[{x}_{i},{y}_{i},{t}_{i},{p}_{i}]$ where ${x}_{i},{y}_{i}$ represent spatial coordinates and ${t}_{i},{p}_{i}$ indicate the time stamp and polarity $(+1 \to ON; -1\to OFF)$ of the events. The image reconstruction is achieved by accumulating the recorded events over the user-defined time slices. For example, in the recorded event data stream over 2 s, the accumulation of 1-ms events yields images every 1/1000 s. This is equivalent to an image acquisition rate of 1000 frames/s. In other words, unlike frame-based cameras, events are not captured at a predefined sampling rate in EB cameras. This allows the user to sample the acquired data at a desired sampling rate based on the relevant flow time scale.

## Coaxial and crossflow air-assist atomizer test facility

**Fig. 2:**
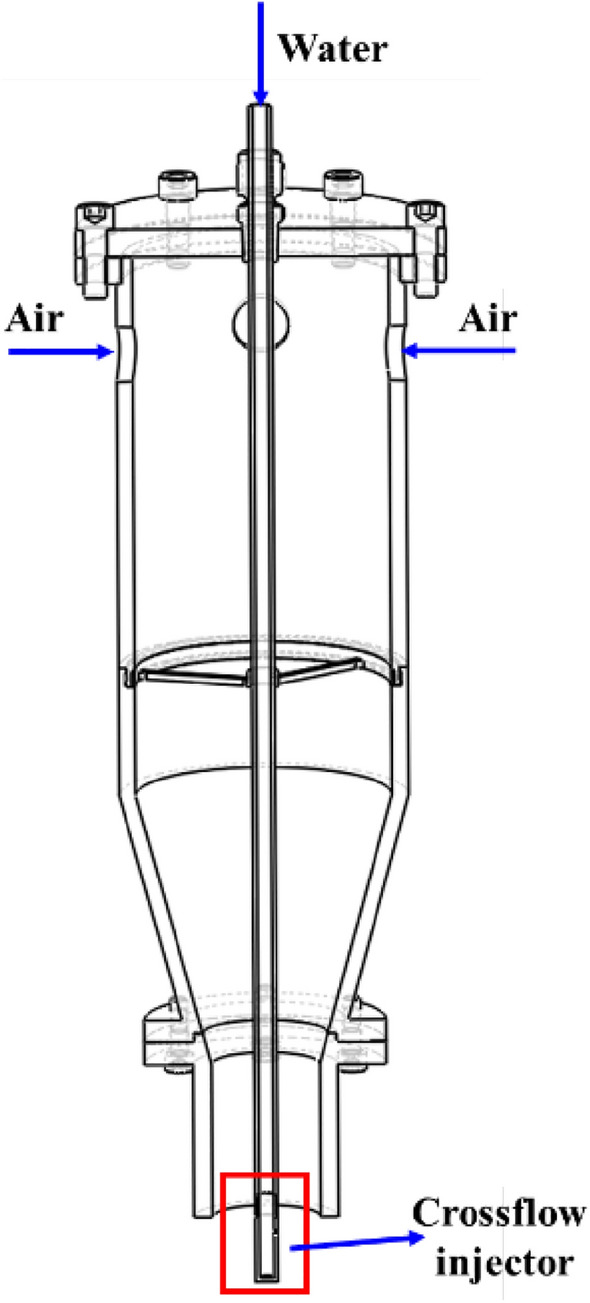
Schematic of the experimental facility of the coaxial/crossflow atomizer

The schematic of the canonical air-assist atomizer test facility used in the present study is shown in Fig. 2**.** The liquid is supplied through a straight concentric tube (ID—5 mm; OD—8 mm) at the top, and the other end is connected to a removable crossflow injector. More details on the atomizer facility can be found in Charalampous et al. (2012). The crossflow injector was closed at the end and had a circular orifice of 1 mm drilled perpendicularly to its centerline. This arrangement injects the liquid jet horizontally into the incoming airflow. With the crossflow nozzle removed, the same test facility acted as a coaxial air-assist atomizer, where the liquid jet was injected parallel to the airflow. During both the crossflow and coaxial atomizer operation, experiments were carried out with three different gas-phase Reynolds numbers ${(Re}_{G})$ representing column/oscillating, bag, and multimodal breakup regimes (Table 1). The liquid flow rate remains constant across all the test conditions. Although the experiments were conducted for three breakup regimes, the bag and multimode breakup regimes are extensively reported here. These two modes are commonly found in the scenario of a liquid jet injected into high-speed turbulent air flows, and they are typically found in many practical sprays (e.g., gas turbine and rocket engine sprays and spray dryers). Moreover, both modes involve complex multiscale interactions with surrounding eddies in turbulent flows, and the associated time scales are much shorter. Thus, the bag and multimode breakup regime characterization mandates time-resolved imaging. This study aims to propose a relatively low-cost imaging device alternative to high-speed cameras; hence, the bag and multimode breakup regime is focused extensively. As briefly outlined in Sect. 2, EB cameras trigger events in relation to the time-varying light intensity caused by the temporal oscillations of the liquid jet. Thus, the columnar breakup mode is chosen only to illustrate how the least oscillations associated with it influence the event generation.

**Table 1:** Test conditions

Coaxial air-assist atomizer			
Test case	Liquid Reynolds number ${(Re}_{L})$	Airflow Reynolds number ${(Re}_{G})$	Breakup regime
A	1431	48,095	Column
B	1431	47,355	Bag
C	1431	54,120	Multimode
*Crossflow atomizer*			
D	2385	53,644	Column
E	2385	52,429	Bag
F	2385	57,503	Multimode

## Time-resolved event-based imaging of liquid jet breakup

**Fig. 3:**
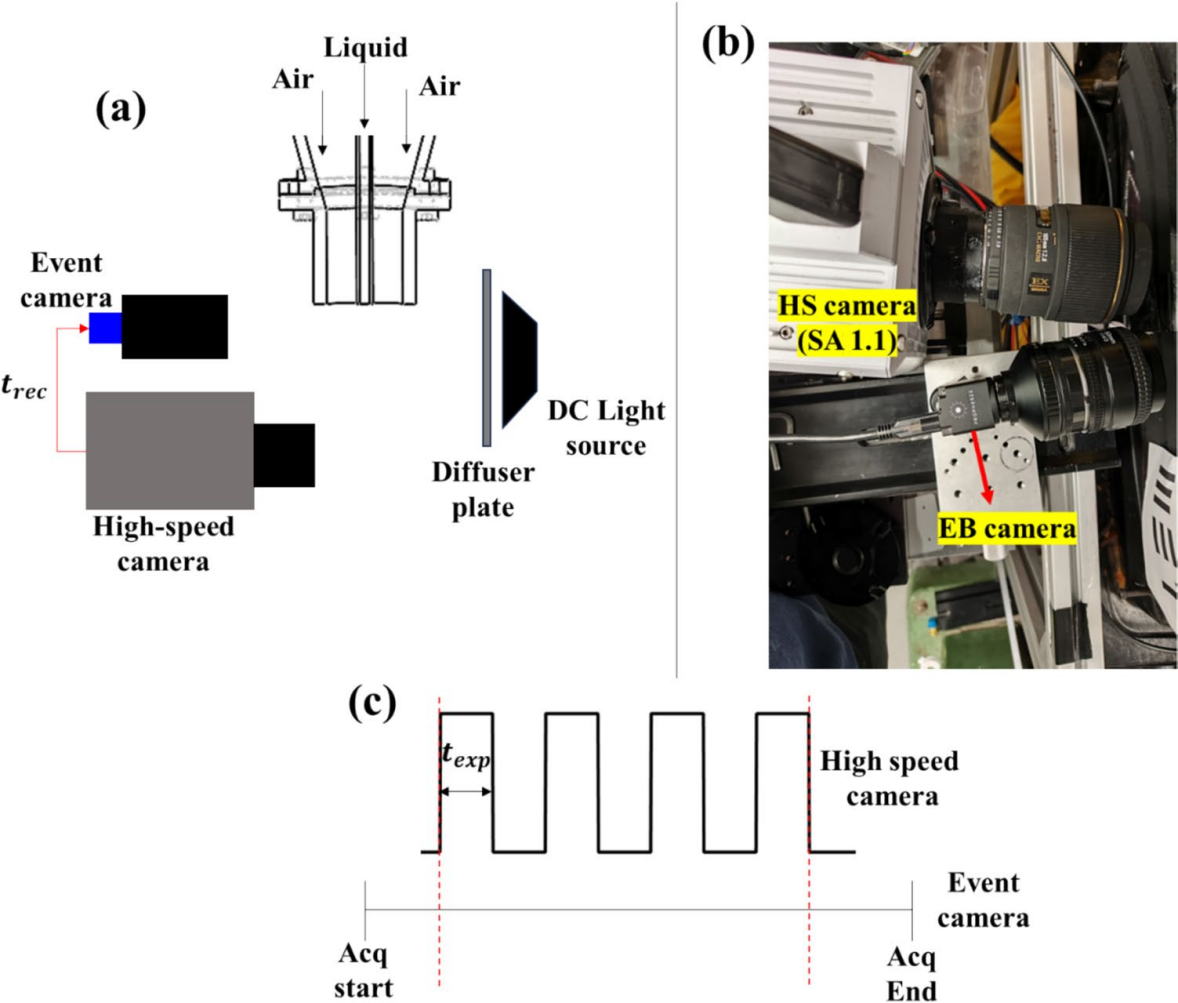
**a** Schematic illustration of the experimental setup of the synchronized imaging of primary atomization using conventional high-speed and event cameras; **b** photograph illustrating mounting of high-speed and EB cameras. **c** Timing diagram of the operation of conventional high-speed and event-based cameras

The optical arrangement involved in the backlight imaging of a liquid jet using an event and high-speed conventional camera is schematically illustrated in Fig. 3. DC-operated continuous wave (CW) white LED light is used for the illumination. In the present work, synchronized measurements were taken with both cameras, allowing us to assess the event camera quantitatively. A Harting 10-pin network cable (09451819002XL) is customized to synchronize both cameras. The event camera (Prophesee—EVK 4; 1980 × 720 pixels) is fitted with a Nikon 50 mm lens (Aperture f/5.6), while the high-speed conventional camera (Photron SA1; 1024 × 1024 pixels) is fitted with a Sigma 105 mm lens (Aperture f/5.6). The field of view for high-speed and event cameras is chosen as 60 mm × 60 mm (MF-136 pixels/mm) and 70 × 40 mm (MF-191 pixels/mm), respectively. The EB camera is much more compact (dimensions—30 mm x 30 mm x 36 mm; weight—70 g) than the Photron SA1 (160 mm x 153 mm × 242.5 mm; weight – 6 kg). In the present study, the HS camera is mounted on a heavy-duty laboratory jack (240 mm x 165 mm), while the EB camera is mounted on a laboratory jack with a small base (132 mm x 80 mm). Although we mounted the EB camera on the laboratory jack in the present study, which is not essential, there could be other simple mounting methods. As illustrated in Fig. 3b, the compactness of the EB camera allows imaging using two cameras almost at the same angle. This feature of the EB camera allows ease of mounting for imaging using multiple EB cameras (e.g., tomo imaging).

The image acquisition rate of the conventional high-speed camera was set at 10,000 fps (sensor resolution—768 × 768 pixels; exposure time—100 µs). A total of 9700 images were acquired, corresponding to a sampling time of 0.97 s. The high-speed camera’s RAM capacity limits the choice of sampling time. The high-speed camera’s recording time cycle ${(t}_{rec})$ is fed into the event camera output data stream $[{x}_{i},{y}_{i},{t}_{i},{p}_{i},{t}_{rec}]$ in the form of 1’s and 0’s, where ‘1’ and ‘0’ correspond to the ON and OFF cycles of the high-speed camera’s recording sequence. This information allowed us to temporally tag the event data stream with the images acquired from the high-speed camera. As the timing diagram illustrates, an event camera does not generate discrete images directly like a high-speed camera; instead, it generates a continuous stream of events. Hence, the event data stream is accumulated over every 100 µs (matching with the exposure time of the HS camera) to reconstruct a total of 9700 images from the recorded event stream over the sampling period of 0.97 s. The setting of optimal accumulation time is crucial in the instantaneous image reconstruction from the recorded event stream from the EB camera. For instance, setting the longer accumulation time leads to streak formation in the images, while smaller accumulation reduces the number of events. Accumulation time in the EB camera is akin to exposure time in the high-speed camera. The exposure time of the high-speed camera is selected based on the flow time scale to avoid streak formation; a similar strategy should also be followed while choosing the accumulation time for image reconstruction from the EB camera. It is worth noting here that the positive feature of the EB camera is that, unlike a high-speed camera, the accumulation time and frame rate are not fixed at the time of acquisition. Hence, the user can set the desired accumulation time during the post-processing stage based on the flow time scale.

Noteworthy is that the generated events are directly transferred to the external storage device instead of temporally stored in the imaging device’s RAM (as is typical for conventional high-speed cameras). For instance, in the present configuration, the choice of sampling time of 0.97 s was limited by the RAM capacity of the high-speed camera. In contrast, technically, an event camera has no such limitation. This feature of the event camera enables real-time high-speed spray imaging even at 10 kHz. More details on comparing image data size and data transfer rate between the high-speed and EB cameras are explained in the following sections.

## Comparison of instantaneous liquid jet structures

**Fig. 4:**
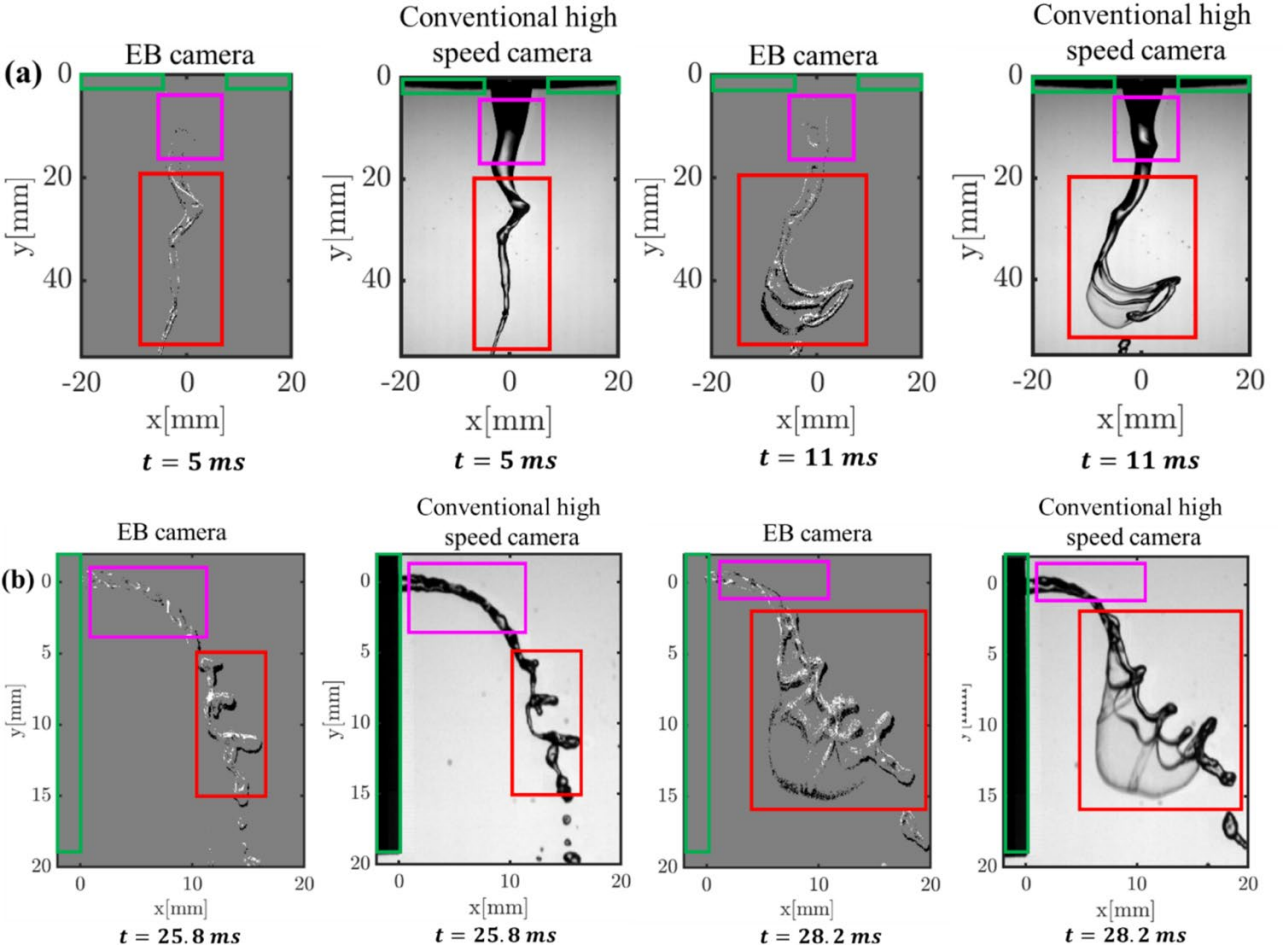
Comparison of reconstructed instantaneous images from the event-based (EB) camera with images acquired from the conventional high-speed camera at different time instants

Figure 4 compares the images acquired simultaneously from the event and conventional high-speed camera during the bag breakup regime (test cases B and E). Here, the black and white color contours on the image reconstructed from the event camera denote the negative and positive events. It is worth noting that the metallic part of the atomizer exit (marked with a green box) is not captured in the reconstructed images of the EB camera.

As the metallic part remains static, inducing no contrast changes, no events are generated in that region. Interestingly, only a few events are witnessed in some regions of the liquid jet despite not being stationary. For instance, the captured events are sparse in the near field of the liquid jet (see areas marked with a pink box in Fig. 4 a, b). In contrast, many events are captured in the far field of the liquid jet (marked with a red box). The reason can be explained as follows: as shown in Fig. 4a, the liquid jet interface near the atomizer exit (pink box) is relatively stable at two-time instants acquired a few milliseconds apart (i.e., t = 5 ms and t = 11 ms), leading to the least changes in the contrast detected by the EB camera pixels at successive time steps. Consequently, fewer events are generated in that region. On the other hand, the liquid jet interface undergoes substantial deformation in the far field (red box) due to the instability, resulting in the detection of strong temporally varying light intensity (contrast changes) on the event camera detector. This triggered more positive and negative events in the region where the liquid jet exhibits strong temporal oscillations. Videos 1 to 6 compare the instantaneous images reconstructed from EB and conventional high-speed cameras across all three breakup regimes (i.e., column, bag, multimode) of coaxial and crossflow atomizers.

It is worth mentioning that the contrast detector threshold can be user-defined to tune the event camera pixels’ response to the light intensity/contrast changes. For example, setting higher threshold values generates events only in regions of the liquid jet where prominent surface deformations occur (e.g., red box). This feature of the event camera is highly beneficial in computing the temporal dynamics of liquid jets in the highly unstable region without interference from the relatively stable region (e.g., the region marked with a pink box). Furthermore, as illustrated in Fig. 4 and Videos 1 to 6, the event generation is directly linked with the contrast changes induced by the moving object; the spatial distribution of the number of generated events in time-resolved temporal space allows the direct identification of the regions where the liquid jet oscillations are prominent. Nevertheless, the EB camera has limitations in imaging liquid structures with negligible surface deformations/oscillations as it triggers only very few events (e.g., column breakup regime, see Video 1). This leads to shortcomings in the whole field characterization of the spray morphology and underpredicts some standard quantities typically measured in sprays (e.g., breakup length). However, illumination with a pulsed light source might address this issue.

## Event generation and data output size

**Fig. 5:**
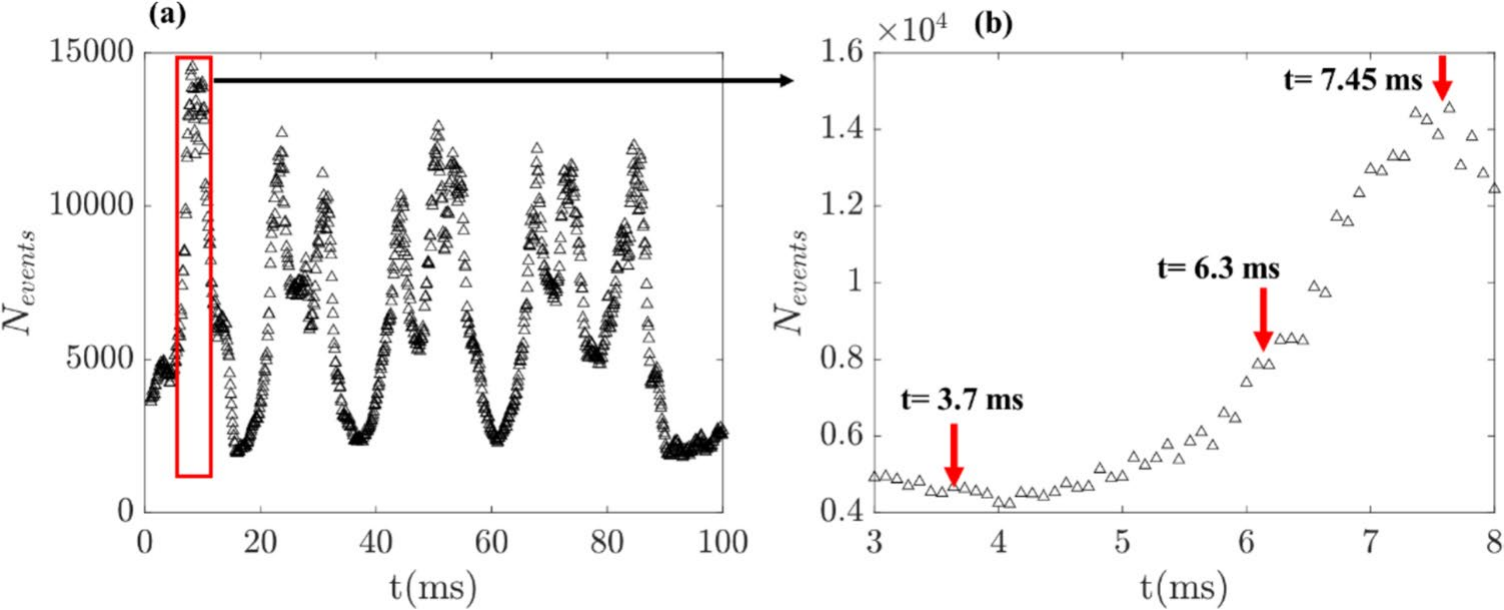
**a**,** b** Illustration of cyclic behavior of event generation in response to the temporally varying liquid jet structure during the bag breakup process in the coaxial atomizer

**Fig. 6:**
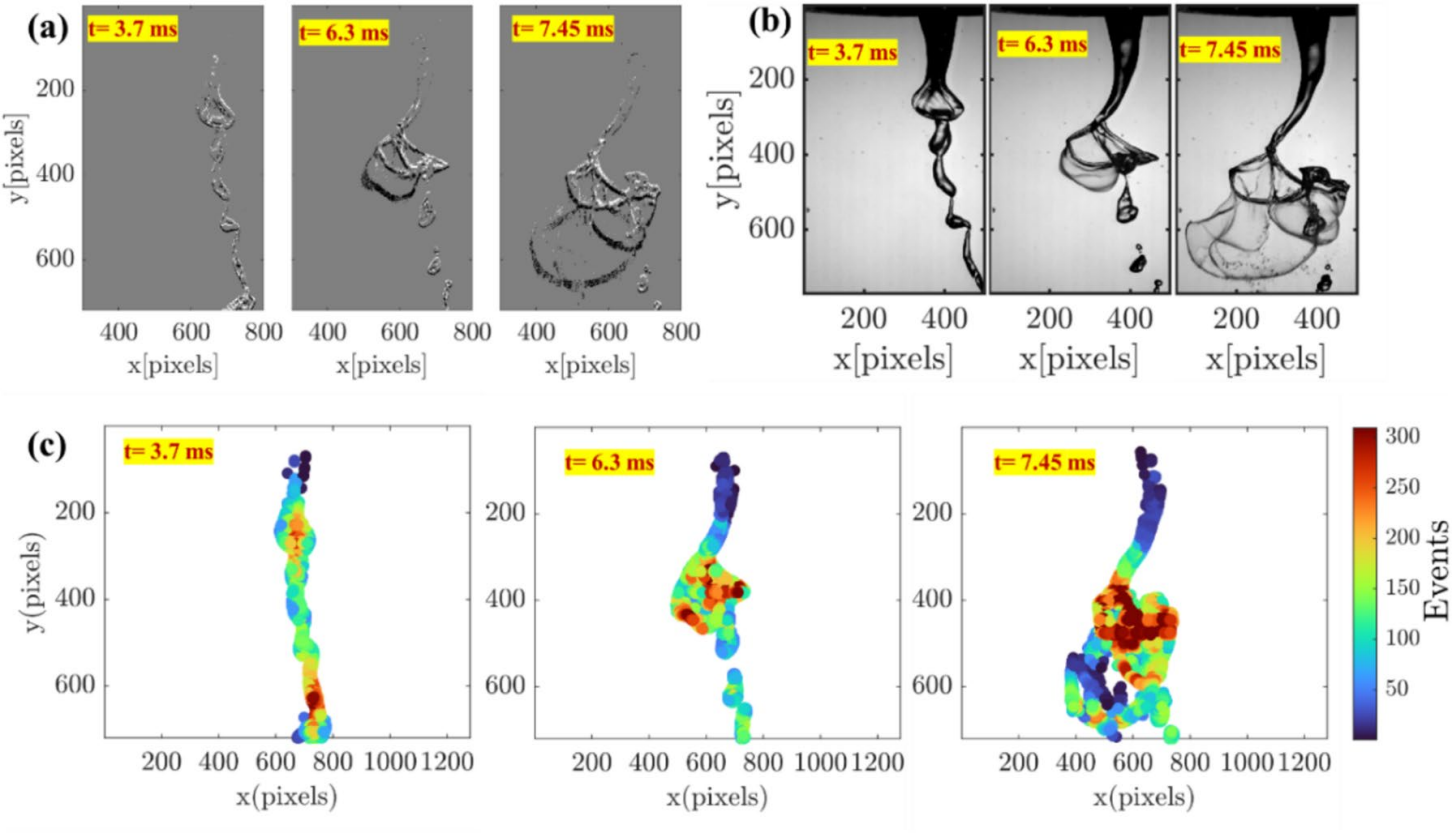
Instantaneous visualization of the liquid jet structure during a bag breakup event in the coaxial atomizer- EB camera (a), conventional high-speed camera (b); c. quantitative illustration of the dynamic change in pixel-wise event generation with temporally changing liquid jet structures

As each pixel of the event camera responds individually to the contrast changes in the scene, the temporally varying liquid jet structures lead to dynamic changes in the number of events (*N*_*events*_) at each time instant, as shown in Fig. 5 a. The output data size of each instantaneous reconstructed image from the event camera solely depends on the *N*_*events*_ during each accumulation period. Therefore, the data size of each reconstructed image from the EB camera differs during the same operating condition, thus significantly reducing data storage requirements. This can be further understood by examining the instantaneous sequences acquired during the liquid jet undergoing bag breakup. Figure 6c illustrates the pixel-wise event generation at different time sequences acquired during the liquid jet bag inflation event. For example, in the sequence acquired before the bag formation (*t* = 3.7 ms), only a few pixels detect the contrast changes (*t* = 3.7 ms in Fig. 6c), generating only ~ 5000 events (see t = 3.7 ms in Fig. 5b). On the other hand, during the liquid jet bag inflation, contrast changes are registered in many pixels (t = 7.45 ms in Fig. 6c), leading to a threefold increase in the number of events *N*_*events*_ ~ 16,000 (see t = 7.45 ms in Fig. 5b).

The final data size of images acquired over the sampling time of 0.97 s for each operating condition is compared for both cameras in Table 2. The data size of the EB camera images is significantly smaller than that of the conventional high-speed camera. Furthermore, it is interesting to note the differences in the file size between different operating conditions. Only a few pixels detected the change in the light intensity during the columnar breakup due to the absence of bag inflation and weaker lateral oscillations of the liquid jet. This leads to fewer positive and negative events; thus, the columnar breakup mode’s data size is smaller than the bag and multimode breakup modes (Table 2). On the other hand, strong lateral jet oscillations and bag inflation during bag and multimode breakup modes lead to the detection of contrast changes in many pixels, triggering many events. Videos 7 and 8 visualize the spatial evolution of event generation in time-resolved temporal space for column and bag breakup modes of coaxial atomizer (test cases A and B). Interestingly, across all the breakup modes, the total number of generated events is higher in the crossflow atomizer than in the coaxial atomizer. This is because, as in the crossflow atomizer, the liquid jet is injected horizontally into the turbulent airflow, leading to a stronger liquid jet oscillation than the coaxial atomizer. Thus, the overall event generation is higher for all the breakup modes in the crossflow atomizer. Conversely to the high-speed camera, the EB camera allows image acquisition at a considerably reduced data transfer rate (Table 3), enabling real-time spray visualization without additional processing. For example, using the simple Python script, we were able to simultaneously visualize and generate time vs. N_events_ histograms at 10 kHz in real time. Furthermore, the temporal evolution of the *N*_*events*_, shown in Fig. 5a, is highly rich in information; for example, since each larger peak of *N*_*events*_ corresponds to a liquid jet bag inflation event, it allows us to identify the exact instant of the bag inflation.

**Table 2:** Comparison of image data file size between high-speed and EB cameras

Breakup mode (test case)	Conventional High-Speed camera		Event-Based camera			
	Coaxial atomizer	Crossflow atomizer	Coaxial atomizer		Crossflow atomizer	
			File size	Total no. of events (∑N_events_)	File size	Total no. of events (∑N_events_)
Column	5.33 GB	5.33 GB	0.087 GB	32 × 10^6^	0.108 GB	38 × 10^6^
Bag breakup	5.33 GB	5.33 GB	0.115 GB	47 × 10^6^	0.138 GB	49 × 10^6^
Multimode	5.33 GB	5.33 GB	0.129 GB	57 × 10^6^	0.145 GB	68 × 10^6^

**Table 3:** Comparison of data transfer rate between high-speed and EB cameras

Breakup mode (test case)	Conventional High-Speed camera		Event-Based camera			
	Coaxial atomizer	Crossflow atomizer	Coaxial atomizer		Crossflow atomizer	
	Data rate (Gb/s)	Data rate (Gb/s)	Data rate (Mb/s)	Event rate (events/s)	Data rate (Mb/s)	Event rate (events/s)
Column	5.49	5.49	90.1	33 × 10^6^	109.9	39 × 10^6^
Bag breakup	5.49	5.49	119.4	48 × 10^6^	131.7	50 × 10^6^
Multimode	5.49	5.49	132.8	58 × 10^6^	150.6	70 × 10^6^

## Comparison of coherent structures of the liquid jet

Dynamic mode decomposition (DMD) was applied to the instantaneous datasets of conventional high-speed and event cameras to compare their performance in capturing the spectral characteristics and coherent structures of the liquid jet characteristics during primary atomization. For brevity, only the results corresponding to the multimode breakup regime of a crossflow atomizer (test case F in Table 1) are presented.

For the DMD implementation, first, the images reconstructed from the EB camera and high-speed camera are arranged in a data matrix ${\varvec{X}}({{\varvec{x}}}_{1},{{\varvec{x}}}_{2},...\boldsymbol{ }{{\varvec{x}}}_{{\varvec{m}}})$**.** As the EB camera records temporal intensity changes in the scene as positive and negative events (+ 1, -1), the image is binarized before the DMD implementation.

The data matrix ${\varvec{X}}({{\varvec{x}}}_{1},{{\varvec{x}}}_{2},...\boldsymbol{ }{{\varvec{x}}}_{{\varvec{m}}})$ is converted into two time-shifted datasets ${{\varvec{X}}}_{1}({{\varvec{x}}}_{1},{{\varvec{x}}}_{2},...\boldsymbol{ }{{\varvec{x}}}_{{\varvec{m}}-1})$ and ${{\varvec{X}}}_{2}({{\varvec{x}}}_{2},{{\varvec{x}}}_{2},...\boldsymbol{ }{{\varvec{x}}}_{{\varvec{m}}})$**.** Next, the linear operator ${\varvec{A}}$ can be represented as follows:

1 $$A{{\varvec{X}}}_{1}={{\varvec{X}}}_{2}$$

Next, the matrix ${\varvec{A}}$ can be represented by taking the pseudo-inverse of ${{\varvec{X}}}_{1}$ as follows:

2 $$A = X_{2} X_{1}^{\dag }$$

The singular value decomposition (SVD) of ${{\varvec{X}}}_{1}$ leads to a pseudo-inverse matrix

3 $${\varvec{X}}_{1}^{\dag } = {\varvec{V}}{{\varvec{\Sigma}}}^{ - 1} {\varvec{U}}^{\user2{*}}$$

4 $${\varvec{A}}={{\varvec{X}}}_{2}{\varvec{V}}{{\varvec{\Sigma}}}^{-1}{{\varvec{U}}}^{\boldsymbol{*}}$$

As the data matrix $({\varvec{X}})$ size is too large, optimal rank ${\varvec{r}}$ obtained based on the higher-order modes is used for the dimensionality reduction. The higher-order modes represent at least 90% of the system’s total energy. For the chosen test case F, the ${\varvec{r}}$ is set to 100 and 117 for the conventional high-speed and event camera datasets, respectively.

The projection of the full matrix ${\varvec{A}}$ based on chosen ${\varvec{r}}$ leads to a low-dimensional matrix $\widetilde{{\varvec{A}}}$ as follows:

5 $$\widetilde{{\varvec{A}}}={{\varvec{X}}}_{2}{{\varvec{V}}}_{{\varvec{r}}}{{{\varvec{\Sigma}}}_{{\varvec{r}}}}^{-1}{{{\varvec{U}}}_{{\varvec{r}}}}^{\boldsymbol{*}}$$

The eigenmode decomposition of $\widetilde{A}$ yields eigenvectors ${\varvec{W}}$ and eigenvalues ${\varvec{\lambda}}$

The corresponding ${\varvec{r}}$ DMD modes can be computed by projecting the eigenvectors ${\varvec{W}}$ onto ${\varvec{U}}$:

6 $${\varvec{\Phi}}={\varvec{U}}{\varvec{W}}$$

where the frequencies $({{\varvec{f}}}_{{\varvec{i}}})$ and growth rates $({{\varvec{\sigma}}}_{{\varvec{i}}})$ can be computed from the eigenvalues

7 $${{\varvec{f}}}_{{\varvec{i}}}=\text{Im}\left(\frac{log{\lambda }_{j}}{2\pi\Delta t}\right)$$

8 $${{\varvec{\sigma}}}_{{\varvec{i}}}=\mathbf{R}\mathbf{e}\left(\frac{log{\lambda }_{j}}{2\pi\Delta t}\right)$$

Finally, the modal amplitudes $({\varvec{C}})$ are obtained from the pseudo-inverse of the DMD mode $({\varvec{\Phi}})$ and initial snapshot:

9 $$C = \Phi^{\dag } x_{1}$$

We recommend that readers refer to the cited literature in this section for more details concerning the various flow instability mechanisms and breakup dynamics of liquid jet in a gas crossflow. A detailed explanation of the various mechanisms is outside the scope of this manuscript.

**Fig. 7:**
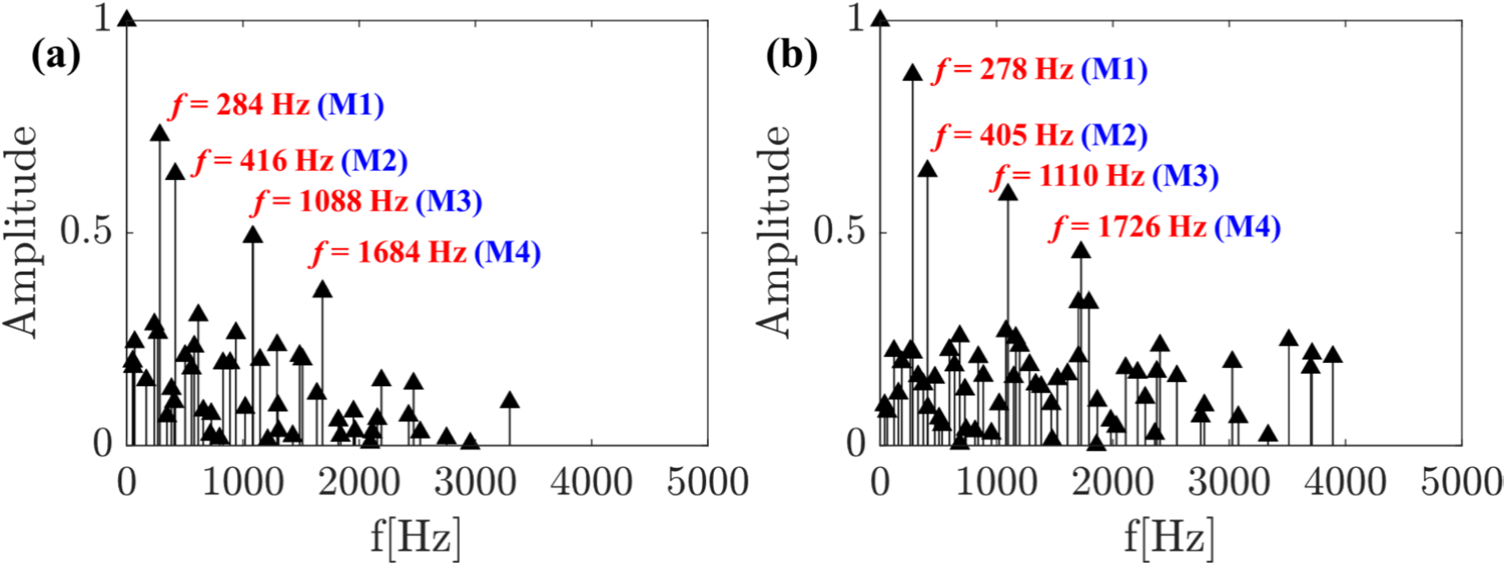
DMD spectrum: **a** high-speed camera; **b** event camera

**Fig. 8:**
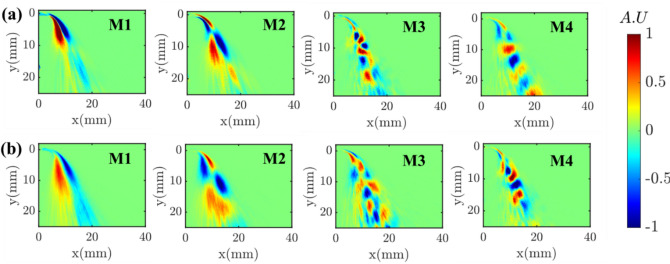
Comparison of dominant DMD spatial modes obtained from a. conventional high-speed camera; b. event-based camera

The DMD frequency spectra obtained from instantaneous datasets of conventional high-speed and event cameras are compared in Fig. 7. The absolute values of the frequencies corresponding to the first four dominant modes (based on amplitudes) are marked, and the corresponding spatial modes are illustrated in Fig. 8. The obtained dominant frequencies are similar for both cameras; nevertheless, a difference of 20–30 Hz is observed, especially at the high-frequency modes (i.e., M3, M4). These discrepancies may be due to differences in the sensor characteristics (e.g., pixel noise levels) between the two cameras. Furthermore, contrary to planar imaging (e.g., PIV), as this is line-of-sight imaging, any slight difference in the depth of field (DOF) between the two cameras could also be the reason for frequency discrepancies. The broadband peaks around 3000–4000 Hz are present in the event camera spectrum, whereas the same is not witnessed in the high-speed camera results. Interestingly, neither of the spatial modes corresponding to 3000–4000 Hz represent any prominent coherent structures, thus confirming noise as the origin. Similar high-frequency broadband noise was observed in the scenario of EB velocimetry applied to the cylindrical wake flow (Willert and Klinner 2022b). However, the origin of such noise in the EB camera is not fully understood yet.

**Fig. 9:**
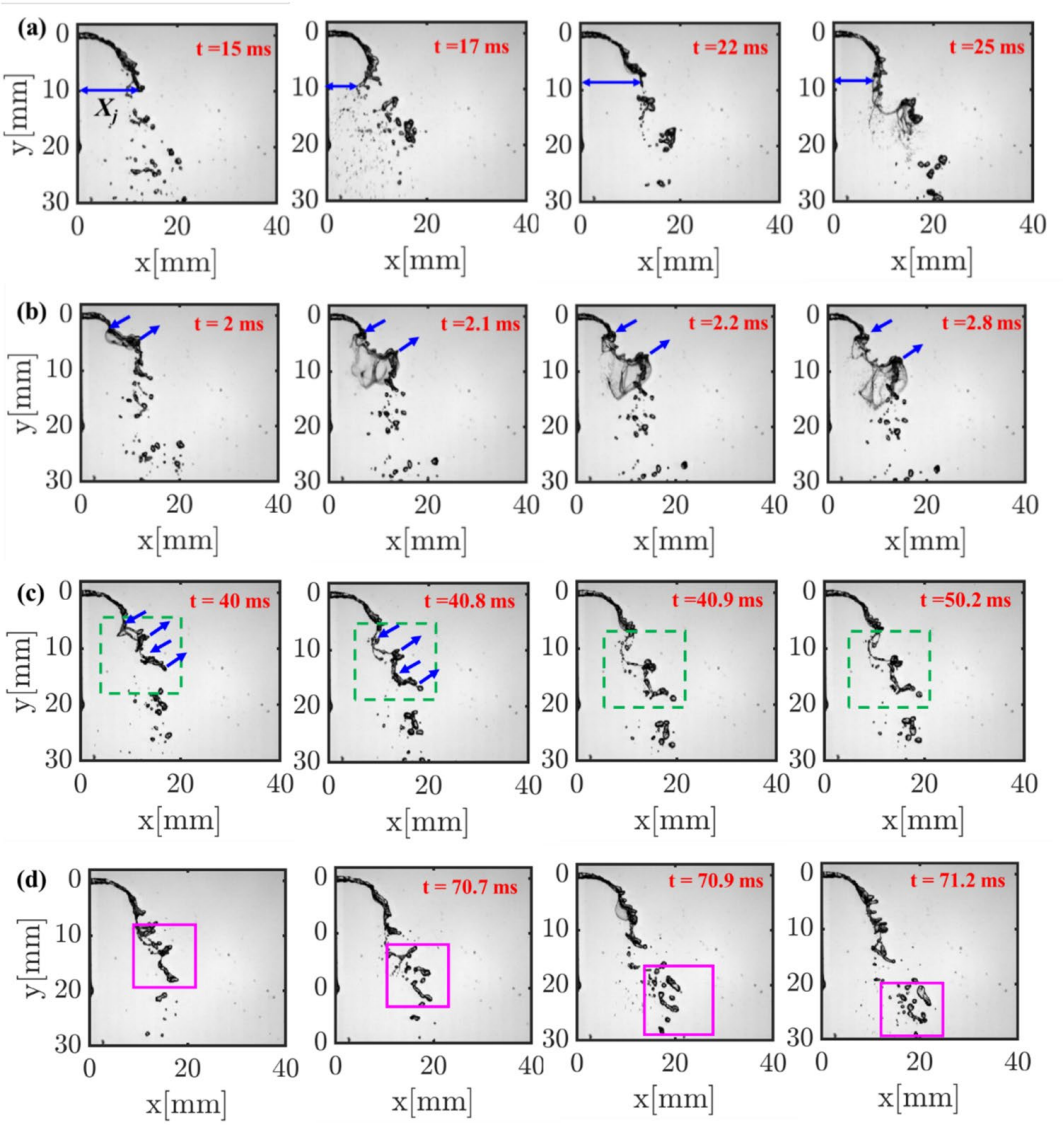
Time-resolved instantaneous sequences illustrating the change in the horizontal position (X_j_) of the liquid jet due to large-scale flapping motion (a), large-scale bending and twisting of the liquid jet (b), small-scale sinuous waves on the liquid jet surface (c); convection of ligaments/droplets in the jet direction (d)

The positive and negative lobes in the spatial mode of M1 represent the time-varying presence of the liquid jet on either side of the liquid jet’s mean trajectory (flapping motion). The time-varying horizontal position of the liquid jet in reference to the injector horizontal axis marked by the parameter *X*_*j*_ (Fig. 9a) demonstrates this behavior. This topology is caused by the flapping instability of the liquid jet and has been identified as the most dominant mode by many researchers (Meyer et al. 2007) (Arienti and Soteriou 2009) (Nair et al. 2019) (Bonnaire and Polifke 2024). Although the positive and negative lobe structures in mode M2 resemble the jet-flapping instability observed in M1, the key difference is that it shows two pairs of lobes, and the signs in each pair appear alternatively in the jet direction. As shown in the instantaneous images, first, the jet bends in the flow direction at an upstream location, followed by twisting of the liquid jet in the opposite direction at a downstream location. This mode is caused by asymmetric sinuous wave formation on the liquid jet interface. Next, the 3rd DMD mode (M3) resembles the similar asymmetric sinuous motion of the liquid jet; however, unlike the M2, the small lobe size represents the small-scale sinuous waves. Such small-scale waves on the liquid jet interface led to a high frequency of ~ 1000 Hz (see M3 in Fig. 7 a, b), much higher than that for M1 and M2. Previous research (Mazallon et al. 1998) (Mazallon et al. 1999) categorized the wavy pattern observed in modes M2 and M3 into long and small wavelength column waves. The instantaneous images illustrated in Fig. 9c display the small-scale sinuous wavy motion of the liquid jet surface, demonstrating the modal structure of M3. Finally, the alternate positive and negative lobes pattern in the jet trajectory (M4 in Fig. 8 a, b) represents the discrete motion of ligaments/primary droplets that formed after the liquid jet breakup. The time scale associated with the displacement of ligaments/droplets is very short due to the convection by the high-speed gas flow, leading to the fast intensity switching (brightness/darkness) in the shadowgraphy images along the jet trajectory/column. Henceforth, the frequency of mode M4 is much higher than that of all the other modes.

The EB camera excludes static or slowly moving structures that could affect the DMD results obtained between the two cameras. However, it should be noted that these static structures are often less coherent and possess weak dynamic features in the flow. In fact, we have implemented the DMD over the entire image and only in the region where the intensity differences are significant due to the strong flapping motion of the liquid jet (for example, the region marked with a red color box in Fig. 5b). The obtained dominant DMD modes are still the same. It is worth mentioning that the EB camera’s intensity change detection threshold can be user-programmed. This lets the user detect only the dynamics of a substantial oscillating portion of the liquid jet by discarding the static/slow-moving moving structures, as they are less significant in representing the dynamics. Furthermore, real-time DMD analysis (e.g., incremental algorithms) of the time-resolved data is emerging in fluid dynamics applications (Zhang et al. 2019), (Schmid 2021), (Schmid 2022). Implementing such techniques to EB camera data is highly possible as it allows the acquisition of time-resolved data at a significantly reduced rate (Table 3). This enables the generation of real-time reduced-order models (ROM), which is essential for flow control applications.

**Fig. 10:**
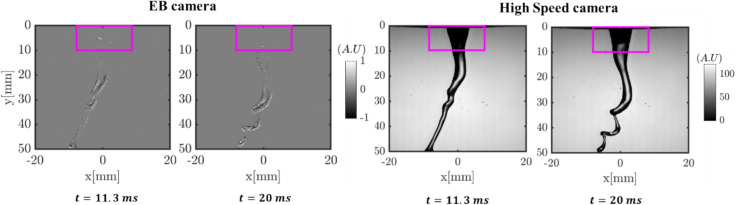
Example of instantaneous images where the intensity difference is negligible in the near field of atomizer across subsequent time sequence

To further assess the EB camera’s potential in modal analysis, DMD is applied only in regions with negligible intensity changes in the consequent frames in high-speed camera images. For instance, in the below sequence corresponding to the column breakup mode of the coaxial atomizer (test case A), the pixel-wise intensity difference between the two consecutive time steps (i.e., from *t* = 11.3 ms to 20 ms) in the near field of the atomizer (marked with a pink color box in Fig. 10) is almost negligible; thus, the EB camera captures few/no events in that region.

**Fig. 11:**
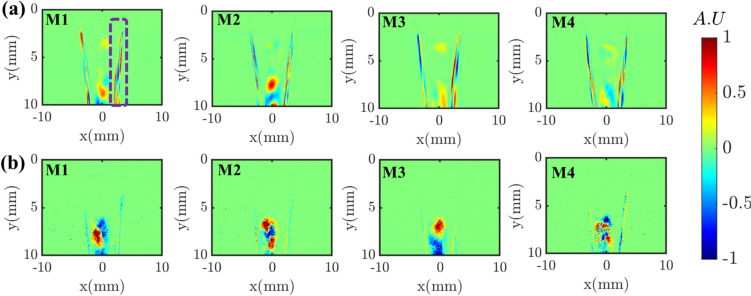
Comparison of dominant DMD spatial modes retrieved from the region where intensity difference is negligible in the consequent time steps **a** conventional high-speed camera; **b** event-based camera

As illustrated in Fig. 11, the obtained DMD modes are not comparable in this case. This is because the high camera captures the absolute intensity, resolving even the tiny oscillations in the liquid jet boundary (as marked with a dashed box in Fig. 11a M1). On the other hand, the EB camera captures the events based on intensity variations, thus undepredicting the spatial DMD modes in the liquid jet boundary where the intensity variations are almost negligible. In these situations, one possible mitigation would be to increase the accumulation time in the EB camera data from 100 µs to 1 ms, potentially increasing the number of events in the reconstructed image. However, this significantly reduces the sampling rate from 10 to 1 kHz. Hence, for fairness, DMD modes between the EB and HS cameras should only be compared when the intensity variations are significant in the subsequent time steps.

## Limitations of event-based imaging in spray applications

Previous studies (Wang et al. 2020) (Willert and Klinner 2022a) have reported the sensor readout saturation issue while capturing scenes that involve too many events (e.g., dense PIV particles). When the EB camera pixel triggers the event, it requests the processing unit to assign the time stamp. The time associated with this process is called readout time, and the time gap between the event detection and time stamp assigning is almost instantaneous. However, let’s say too many events are instantaneously generated at a time across many pixels (e.g., bag breakup); the processing unit no longer reads all the events simultaneously, although they were generated instantly. This introduces a delay in assigning the time stamps, eventually influencing the timing precision. This scenario is referred to as readout saturation. Unlike CMOS/CCD sensors, the readout saturation in EB cameras is essentially caused by the event rate. Hence, it is sometimes referred to as event saturation. The maximum event rate threshold of the EB camera used in this study is ~ 80 × 10^6^ events/s; thus, exceeding this threshold results in event saturation. It should be noted that the event rate handling capacity of the EB cameras is continuously improving; for example, the maximum event rate threshold of the previous version is 50 × 10^6^ events/s.

**Fig. 12:**
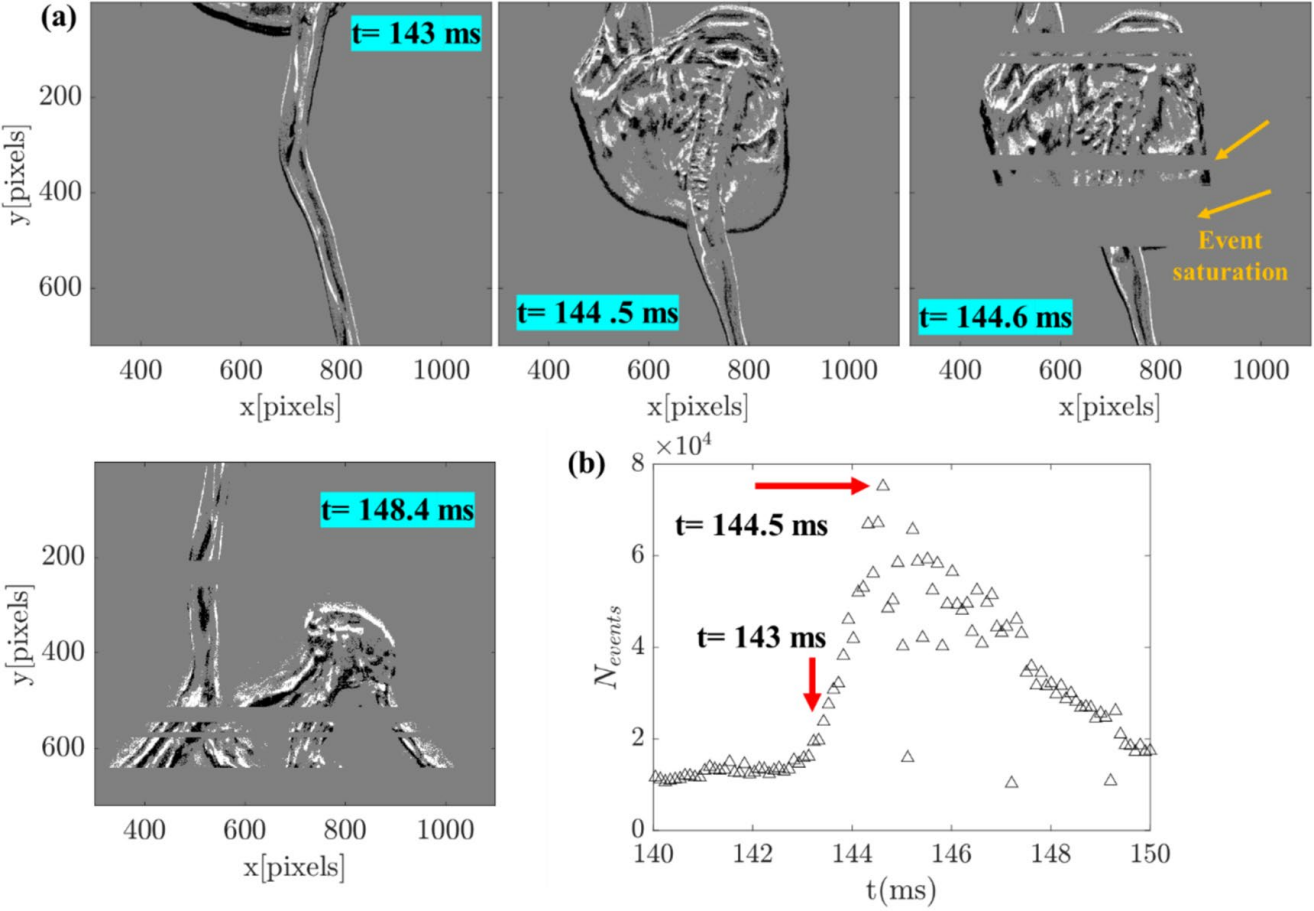
**a** Instantaneous images illustrating the event saturation behavior observed during high-magnification imaging of the bag breakup process in the coaxial atomizer (test case B); **b** time evolution of event generation

Magnified imaging with a field of view of 25 × 18 mm (MF—40 pixels/mm) is performed to assess this phenomenon in spray applications further. As shown in Tables 2 and 3, contrary to columnar breakup mode, bag inflation and strong lateral oscillations triggered events across several pixels during bag and multimode operations. Thus, high-magnification imaging was carried out with only these two modes. The time sequence of the single-bag breakup captured with magnified viewing and the corresponding number of events (*N*_*events*_) is illustrated in Fig. 12. As the bag inflates (Fig. 12a; *t* = 144.5 ms), approximately 70% of the pixels are exposed, resulting in a sharp rise in the *N*_*events*_ ~ 75,000, which is approximately five times higher than the *N*_*events*_ ~ 14,000 obtained during similar bag inflation captured with low-magnification imaging (Fig. 5 a, b; *t* = 7.45 ms). During this high-magnification imaging, the event rate hits ~ 110 × 10^6^ events/s, much higher than the maximum threshold (80 × 10^6^ events/s) of the EB camera used in this study. Due to the sharp rise in event generation at t = 144.5 ms, a temporal loss of events occurred in the subsequent time sequences. The event loss manifests as horizontal patches in the reconstructed images (marked with yellow arrows in *t* = 144.6 ms; also see Video 9). Noticeably, the events lost at *t* = 144.6 ms will be registered in the arbiter in the subsequent time steps. This introduces the delay between the actual occurrence of the event and the time assigned by the EB camera’s processing unit. The delay could be of the order of several micro to milliseconds, and it is challenging to quantify this. For example, the actual occurrence of the reconstructed image from the EB camera shown at *t* = 148.4 ms was *t* = 145.3 ms (confirmed with the images acquired simultaneously with a high-speed camera). Hence, the loss of temporal resolution caused by the event saturation cannot be quantified unless additional online monitoring is employed simultaneously with the EB camera. Consequently, the event data shown in Fig. 12 cannot be reliably processed to extract features like temporal displacement.

Reducing the region of interest (ROI) on the sensor, i.e., the number of pixels used to monitor the intensity changes, can increase the event count per pixel as the total number of events is significantly reduced. This is the reason why the total number of events is within the maximum threshold during the low-magnification imaging of the bag and multimode breakup regimes (see Table 2), where only 50% of the pixels were used. Furthermore, pulsed illumination with shorter pulse widths can mitigate the event saturation issue. For instance, in the above-discussed example, the LED light source pulsing at 10 kHz with a pulse duration of 1 to 2 microseconds will generate events only when the light pulse is ON. Moreover, the pulsed illumination will also help detect slow-moving structures.

The following summary provides the advantages and disadvantages of the EB camera in the context of spray imaging.

Advantages.

- Unlike the traditional high-speed camera, Event-based imaging involves a considerably reduced data rate, allowing real-time spray visualization without additional post-processing.
- The EB camera’s reduced data rate feature allows the implementation of data-driven techniques like DMD in real time. Such implementation allows the generation of real-time reduced-order models necessary for flow control applications.
- The intensity changes detection threshold can be user-programmed, allowing only the detection of a strong oscillating portion of the liquid jet. This could help to extract flow dynamics corresponding to these structures by isolating dynamics of the quiescent portion of the liquid jet.
- Contrary to conventional high-speed cameras, the flexibility of varying the accumulation time during the post-processing stage allows the user to extract information on the trajectories of the droplets with high temporal resolution.

Limitations / Disadvantages.

- In the scene involving quiescent and oscillating structures, the image reconstructed with a shorter accumulation time excludes slow-moving structures. Such exclusions limit the measurement of standard quantities in sprays (e.g., breakup length). This can be overcome by increasing the accumulation time. However, this induces streak formation by the fast-moving structures.
- The event saturation issue is the major limiting factor in spray imaging at high magnification (especially for bag and multimode breakup regimes). The maximum event rate threshold of the EB camera sensors is continuously improving, which could overcome this shortcoming.

## Conclusions

The present work demonstrates an alternative approach to conventional high-speed imaging using a low-cost event-based camera. The application is focused on time-resolved (10,000 frames/sec) imaging of the primary atomization region in canonical air-assist atomizers. Synchronized experiments were conducted to assess the performance of both cameras quantitatively. The performance of the EB camera is accessed across three widely used spray breakup regimes: column, bag, and multimode. The evolution of event generation rates of the EB camera across these breakup modes is meticulously analyzed, as they are the prime factors in determining the final image data size. The absence of bag inflation and weaker jet oscillations during columnar breakup mode triggered events in only a few pixels. Hence, its data size is smaller than that of other modes. Subsequently, DMD was implemented to compare the coherent structures in the primary atomization zone obtained from the images of both cameras. The obtained DMD modes from both cameras are comparable, highlighting the potential of low-cost event-based cameras in extracting coherent structures and their spectral contents of the liquid jet breakup characteristics. However, it should be noted that DMD modes retrieved from both cameras are comparable only when the change in light intensity is significant in the consequent time steps. Overall, the present study shows the potential of EB cameras in high-speed spray imaging at reduced capital cost. Nevertheless, issues like the arbiter saturation at high-magnification spray imaging must be addressed.

## Supplementary Information

Below is the link to the electronic supplementary material.

Supplementary file1 (AVI 4740 KB)

Supplementary file2 (AVI 2972 KB)

Supplementary file3 (AVI 4181 KB)

Supplementary file4 (AVI 1540 KB)

Supplementary file5 (AVI 2753 KB)

Supplementary file6 (AVI 2350 KB)

Supplementary file7 (AVI 3865 KB)

Supplementary file8 (AVI 4105 KB)

Supplementary file9 (AVI 4110 KB)

## Data Availability

No datasets were generated or analyzed during the current study.
